# Supplementary material for: Reduced dengue incidence following deployments of Wolbachia-infected Aedes aegypti in Yogyakarta, Indonesia: a quasi-experimental trial using controlled interrupted time series analysis
Source: Gates Open Res. 2020 May 11;4:50. [Version 1] doi: 10.12688/gatesopenres.13122.1 (PMC7403856; doi:10.12688/gatesopenres.13122.1)
Supplement: Peer review history

# Peer review: Reviewer response for version 1

**DOI:** 10.21956/gatesopenres.14303.r29161

**Published:** 2020-08-04

**Authors:** Basile Kamgang

**Affiliations:** 1 Department of Medical Entomology, Centre for Research in Infectious Diseases, Yaoundé, Cameroon

This paper presents interesting works which demonstrated that Ae. aegypti infected with Wolbachia contribute to reducing the incidence of dengue. However, I have some minor comments that I would wish to have the point of view of the authors.

1. Mosquitoes blood-fed by human volunteers. There is no possible alternative measure? What was the compensation for humans? There is no risk of infection for human volunteers with wild strains of *Ae. aegypti* since the vertical transmission of dengue was documented? In addition, Yogyakarta is an endemic area for dengue.
2. Figure 2 diagnostic doses of insecticides used notably 0.75% permethrin and 0.05% deltamethrin are not compatible with those recommended by WHO for *Aedes* mosquitoes (0.025% permethrin and 0.03% deltamethrin).
3. Both strains of Ae. aegypti used are resistant to pyrethroid, the authors think that the result could be the same with the susceptible pyrethroid strains? What could be the potential implication of insecticide resistance and vector competence?
4. Do the authors know whether Wolbachia is efficient to block dengue virus alone or in association with other bacteria?
5. “All volunteer blood-feeders were a febrile and free of clinical signs or symptoms of any arbovirus infection both at the time of blood-feeding and for three days thereafter”. What is the prevalence of the asymptomatic cases of dengue in the area? For me, I think all the volunteers should be screened by molecular tools to detect any presence of the dengue virus before the experiment.

Is the work clearly and accurately presented and does it cite the current literature?

Yes

If applicable, is the statistical analysis and its interpretation appropriate?

Yes

Are all the source data underlying the results available to ensure full reproducibility?

Yes

Is the study design appropriate and is the work technically sound?

Yes

Are the conclusions drawn adequately supported by the results?

Yes

Are sufficient details of methods and analysis provided to allow replication by others?

No

Reviewer Expertise:

Aedes-borne diseases, Insecticide resistance in arbovirus vectors, vector competence and bionomics of Aedes

I confirm that I have read this submission and believe that I have an appropriate level of expertise to confirm that it is of an acceptable scientific standard.

# Peer review: Reviewer response for version 1

**DOI:** 10.21956/gatesopenres.14303.r29159

**Published:** 2020-07-28

**Authors:** Roberto Barrera

**Affiliations:** 1 Entomology and Ecology Activity, Dengue Branch, Centers for Disease Control and Prevention, San Juan, Puerto Rico

This study compared dengue disease incidence in areas where the authors released and established local populations of ***Wolbachia***-infected ***Aedes aegypti***and a nonintervention area, before and after releases. For a study without replication, it seems that the two study areas were rather comparable in terms of dengue cases before the intervention, which speaks in favor of interpreting the results after the intervention. As such, the authors reported important reduction in dengue cases as a result of the intervention. The results of the establishment of **Wolbachia** in **Ae. aegypti** at very high proportions after the releases, add support to the inferred mechanism by which the intervention was successful (blocking virus transmission). Data were properly analyzed, results were clearly presented, and the conclusions are supported by the data.

 Minor comments:

 It would help understand the experimental setup if more details were given on the ecological and social conditions of the intervention and control areas.

 Describe rationale for the number of monitoring BG traps and explain the difference in trap density between treatment (15.7 BGs/Km2) and control (3.6 BGs/Km2) sites.

 Why the wMel strain tended to be more resistant to insecticides than the wild-type?

 Were there any concurrent vector control actions during the study? Have you considered repeating insecticide resistance testing over time? What types of interactions and how traditional or official mosquito control actors reacted to this project?

 Was there any correlation between dengue incidence in treatment and control sites during the two years after treatment?

 Can you describe how residents of the intervention sites interpreted or understood the mechanism by which releasing these **Wolbachia**-infected mosquitoes acted to suppress dengue virus transmission?

Is the work clearly and accurately presented and does it cite the current literature?

Yes

If applicable, is the statistical analysis and its interpretation appropriate?

Yes

Are all the source data underlying the results available to ensure full reproducibility?

Yes

Is the study design appropriate and is the work technically sound?

Yes

Are the conclusions drawn adequately supported by the results?

Yes

Are sufficient details of methods and analysis provided to allow replication by others?

Yes

Reviewer Expertise:

Medical Entomology

I confirm that I have read this submission and believe that I have an appropriate level of expertise to confirm that it is of an acceptable scientific standard.
